# Enzyme-Nanoporous Gold Biocomposite: Excellent Biocatalyst with Improved Biocatalytic Performance and Stability

**DOI:** 10.1371/journal.pone.0024207

**Published:** 2011-09-02

**Authors:** Xia Wang, Xueying Liu, Xiuling Yan, Peng Zhao, Yi Ding, Ping Xu

**Affiliations:** 1 State Key Laboratory of Microbial Technology, Shandong University, Jinan, People's Republic of China; 2 School of Chemistry and Chemical Engineering, Shandong University, Jinan, People's Republic of China; 3 State Key Laboratory of Microbial Metabolism and School of Life Sciences and Biotechnology, Shanghai Jiao Tong University, Shanghai, People's Republic of China; The University of Manchester, United Kingdom

## Abstract

**Background:**

Applications involving biomolecules, such as enzymes, antibodies, and other proteins as well as whole cells, are often hampered by their unstable nature at extremely high temperature and in organic solvents.

**Methodology/Principal Findings:**

We constructed enzyme-NPG biocomposites by assembling various enzymes onto the surface of nanoporous gold (NPG), which showed much enhanced biocatalytic performance and stability. Various enzymes with different molecular sizes were successfully tethered onto NPG, and the loadings were 3.6, 3.1 and 0.8 mg g^−1^ for lipase, catalase and horseradish peroxidase, respectively. The enzyme-NPG biocomposites exhibited remarkable catalytic activities which were fully comparable to those of free enzymes. They also presented enhanced stability, with 74, 78 and 53% of enzymatic activity retained after 20 successive batch reactions. Moreover, these novel biocomposites possessed significantly enhanced reaction durability under various thermal and in organic solvent systems. In a sample transesterification reaction, a high conversion rate was readily achieved by using the lipase-NPG biocomposite.

**Conclusion/Significance:**

These nano-biocomposite materials hold great potential in applications such as biosensing, molecular electronics, catalysis, and controlled delivery.

## Introduction

The encapsulation of biomolecules, such as enzymes, antibodies, and other proteins as well as whole cells, on porous materials is an important approach to stabilize the biological components in what is often an unnatural environment while retaining their functions and activities [1]–[7]. However, there still exist a series of technical bottlenecks for practical applications of immobilized enzymes, such as low catalytic activity, restricted mass transfer, enzyme leaching upon reuse, and sophisticated and expensive synthesis procedures. Taking into consideration all these factors, nanoporous gold (NPG), fabricated by a simple dealloying method, was selected here as a support for enzyme immobilization due to its unique physicochemical properties [8]–[10]. NPG has tunable pores at a nanometer scale that could fit proteins with different molecular weights and dimensions and offer the possibility of adsorbing or entrapping biomolecules within the pores as well as on the external surfaces depending on the nature of the proteins. In addition, NPG is intrinsically a nanostructured bulk material, thus it can be easily employed and recovered for reuse. Moreover, NPG has a biocompatible and active surface, which offers the opportunity for covalent binding through for example the well known thiol-based self-assembled monolayer technology [11].

## Materials and Methods

### Chemicals

4-Nitrophenyl palmitate, p-nitrophenol, pyrogallol, lipase (Aldrich 534641 from *Pseudomonas cepacia*), catalase, and horseradish peroxidase (HRP) were purchased from Sigma-Aldrich (St. Louis, USA).

### Enzymes immobilization

Nanoporous gold was made by chemically dealloying AgAu alloy foils (Ag_78_Au_22_ at.%, 25 µm in thickness, purchased from Changshu Noble Metal Company, China) in concentrated HNO_3_ at room temperature for a certain period of time [12]. The size and specific surface are ca. 35 nm and ca. 14 m^2^ g^−1^, respectively. For enzyme immobilization on NPG, 1 ml of enzymes solution (1.0 mg ml^−1^ of lipase, 1.0 mg ml^−1^ of catalase, and 0.01 mg ml^−1^ of HRP, respectively) was mixed with 18 mg NPG. Then the mixture was incubated at 4°C. After incubation, the supernatant was removed by centrifugation (5,000× *g* for 5 min), and the enzyme-NPG biocomposite was washed five times with distilled water before measuring the activity of the immobilized enzyme. The amount of immobilized enzyme was determined by Bradford protein assays [13].

### Activity assays

The activity of immobilized or free lipase was determined by measuring its initial hydrolysis rate of 4-nitrophenyl palmitate at 40°C according to a previous literature [14]. One unit (U) of lipase activity is defined as the amount of lipase which catalyzes the production of 1 µg *p*-nitrophenol under the experimental conditions. For determining HRP activity, we inserted the biocomposite in a medium containing pyrogallol at 5% (w/v) and hydrogen peroxide at 0.5 (w/v), while absorbance at 420 nm was monitored with time, as described elsewhere [15]. The activity of catalase was determined spectrophotometrically by indirect measurement according to a previous literature [16]. The enzymatic reaction was carried out at 25°C, then stopped with 2.7 ml of 50 mM ammonium molybdate and the yellow complex of molybdate and hydrogen peroxide was measured at 405 nm.

### Characterization

The microstructures of NPG and NPG bound to lipase were characterized using a JEOL JSM-6700F field emission scanning electron microscope (SEM), equipped with an Oxford INCA x-sight energy-dispersive X-ray spectrometer for compositional analysis. X-ray photoelectron spectroscopy (XPS) was taken with ESCALAB 250 (Thermo-Fisher Scientific, UK), using monochromated Al Kα excitation at pass energy of 150 eV for survey spectra and 20 eV for core lever spectra. The bonding energies were referenced to the C 1s BE at 284.6 eV. Lipase-loaded NPG and bare NPG were stained with a solution of fluprescein isothiocyanate dextran in carbonate buffer (0.5 mol l^−1^, pH 8.0) for 30 min at 30°C, and then washed with distilled water. After staining, the samples were characterized using an Eclipse 80i/90i Microscope (Nikon, Japan). The surface area of NPG used was measured with Quadrasorb SI-MP (Quantachrome Instrument) using the BET method.

### Thermal and organic solvent stabilities

The thermal stability of the lipase-NPG biocomposite was examined by measuring the residual activity at 40°C after incubation in Tris-HCl buffer (50 mM, pH 8.0) for 30 min at 50, 60, and 70°C. To test their durability toward organic media, free and lipase-NPG biocomposite samples were exposed to an aqueous solution containing 50% (v/v) benzene, ethyl acetate, and chloroform, at 30°C for 12 h on a rotary shaker at 150 rpm. And then the residual activity was detected.

### Enzymatic transesterification reaction

The transesterification reaction was carried out in 100-ml flasks at 40°C on a rotary shaker at 150 rpm. The composition of the reaction mixtures was 2.0 ml of refined soybean oil, immobilized lipase (2.7 mg of lipase) or free lipase (2.7 mg), 1.0 ml distilled water and 167 µl of methanol (added twice, the final volume ratio of oil∶methanol is 6∶1). After 24 h reaction, the methyl esters were extracted in n-hexane for gas chromatographic analysis [14].

## Results

### Construction of enzyme-NPG biocomposite

**Figure 1:**
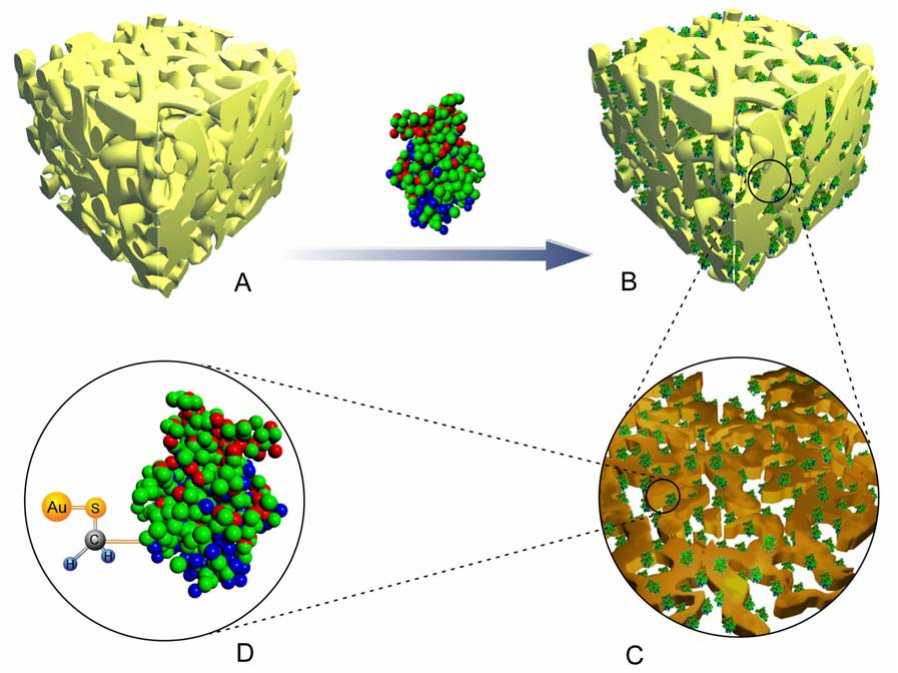
Schematic illustration of lipase immobilization onto NPG.

To investigate the potential of NPG for bio-macromolecule immobilization, several model enzymes such as lipase, catalase and HRP with different molecular weights were employed in this study. The dimensions of lipase, catalase and HRP revealed by their crystal structures as reported are in the range of 4–10 nm as shown in Table 1 [17]–[19]. According to theoretical calculations, maximum stabilization of a protein can be achieved by adsorption within spherical cages whose diameter is 2–6 times of the dimension of the native molecule [5]. Therefore, NPG with a pore size of ca. 35 nm was used here for enzyme immobilization. The procedure of the enzyme adsorption onto NPG can be illustrated in Figure 1.

**Table 1:** Enzyme properties and immobilized amounts on NPG.

Enzyme	Mw (kDa)	Size of enzyme (nm)	Enzyme loading (mg g^−1^)
Lipase	25	5	3.6
Catalyse	250	10	3.1
HRP	40	4	0.8

### Characterization of enzyme-NPG biocomposite

**Figure 2:**
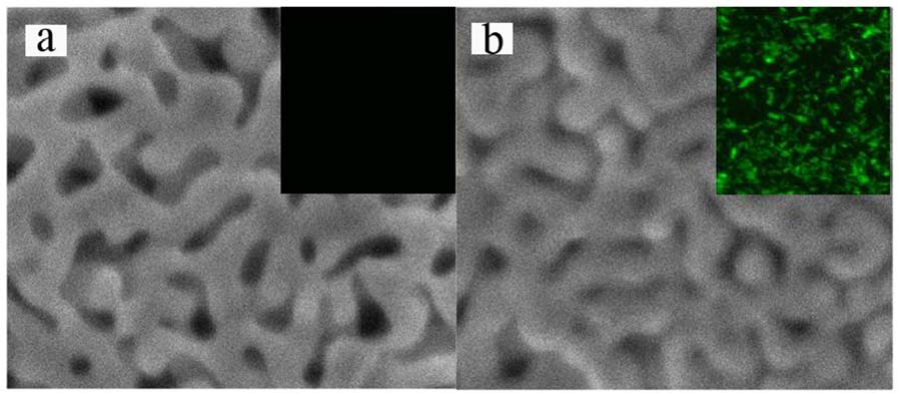
SEM images of NPG before (a) and after (b) lipase loading. Insets are the respective fluorescence microscope images.

Samples of NPG before and after the enzyme loading were characterized using scanning electron microscopy. Compared with the bare NPG (Figure 2a), the enzyme-NPG biocomposite looks a little blurred due to its poorer conductivity (Figure 2b). Smaller pore sizes and relatively smoother surface morphology were found for the enzyme-NPG biocomposite, indicating preferential immobilization of the enzyme molecules over the ligament site with high radial curvatures. A similar phenomenon was observed previously when modifying the NPG surface with Pt [20]. Meanwhile, a homogeneous fluorescence distribution on the enzyme-NPG biocomposite was observed after staining with fluorescein isothiocyanate dextran by fluorescence microscopy analysis (inset in Figure 2b), while no fluorescence was observed on the bare NPG treated under the same conditions (inset in Figure 2a). The loadings of lipase, catalase and HRP are 3.6, 3.1 and 0.8 mg g^−1^, respectively (Table 1). For HRP, the lower loading may be due to the lower enzyme concentration.

### Catalytic performance of enzyme-NPG biocomposite

**Figure 3:**
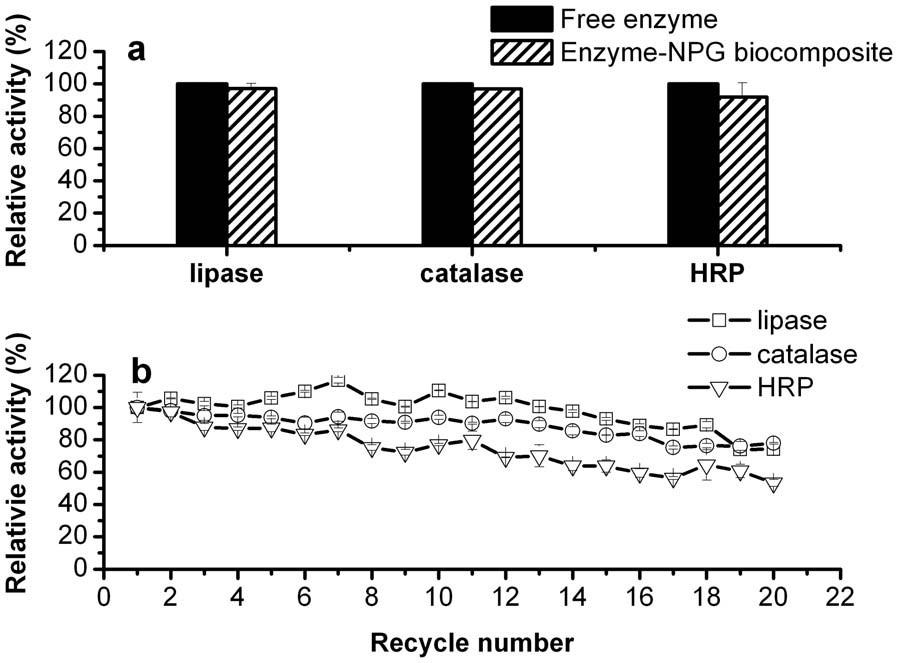
Activities (a) and reusabilities (b) of the enzyme-NPG biocomposites.

As expected, the enzyme-NPG biocomposites demonstrated remarkable catalytic performance and stability. There were nearly no decreases in catalytic activities upon immobilization for the enzyme-NPG biocomposites as shown in Figure 3a. These results indicated significant improvement of catalytic activities in our systems as compared with the less biocompatible ones [21]–[23]. This should be associated with NPG's excellent biocompatibility, active surface, and high conductivity, which are very important for enzyme immobilization. In addition, the enzyme-NPG biocomposites exhibited remarkable reaction stability (Figure 3b). For example, no decrease in activity was observed by the lipase-NPG biocomposite after recycling for ten times (Figure 3b). Moreover, there is still 74% of activity after 20 successive batch reactions. While for the catalase-NPG and HRP-NPG biocomposites, their activities decreased by only ca. 7 and 23% after ten cycles, respectively. After 20 successive batch reactions, the catalase-NPG and HRP-NPG biocomposites could still maintain 78 and 53% of activities, respectively.

### Stability of lipase-NPG biocomposite under different conditions

**Figure 4:**
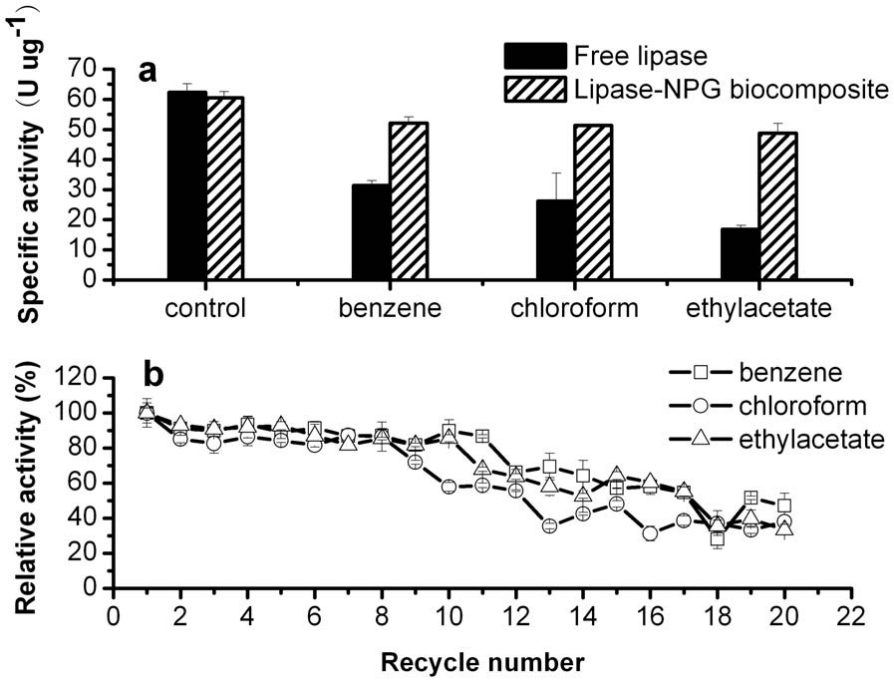
Tolerance stabilities of free lipase and the lipase-NPG biocomposite to organic solvents (a), and reusability of the lipase-NPG biocomposite after treated by organic solvents (b).

The stability of the lipase-NPG biocomposite under various thermal and in organic solvent systems was investigated. The logarithm of the partition coefficient between 1-octanol and water (log *P*) is widely used to characterize molecular lipophilicity and hydrophobicity [24]. In order to study the tolerance of the lipase-NPG biocomposite to organic solvents, benzene (log *P* is 2.0), ethyl acetate (log *P* is 0.68), and chloroform (log *P* is 2.0) were employed. As shown in Figure 4a, for free lipase, only 50, 27, and 42% of initial activity were observed after treatment with benzene, ethyl acetate, and chloroform, respectively. In contrast, the lipase-NPG biocomposite exhibited much enhanced stability in organic solvents with 86, 81, and 85% of activity retention after treatment in the same solvents. No significant decrease in catalytic activity was observed for the lipase-NPG biocomposite after 10 cycles under the test conditions (Figure 4b). Even after 20 recycles, there were sill 47, 33 and 38% activity retention for the lipase-NPG biocomposite treated by benzene, ethyl acetate, and chloroform, respectively.

**Figure 5:**
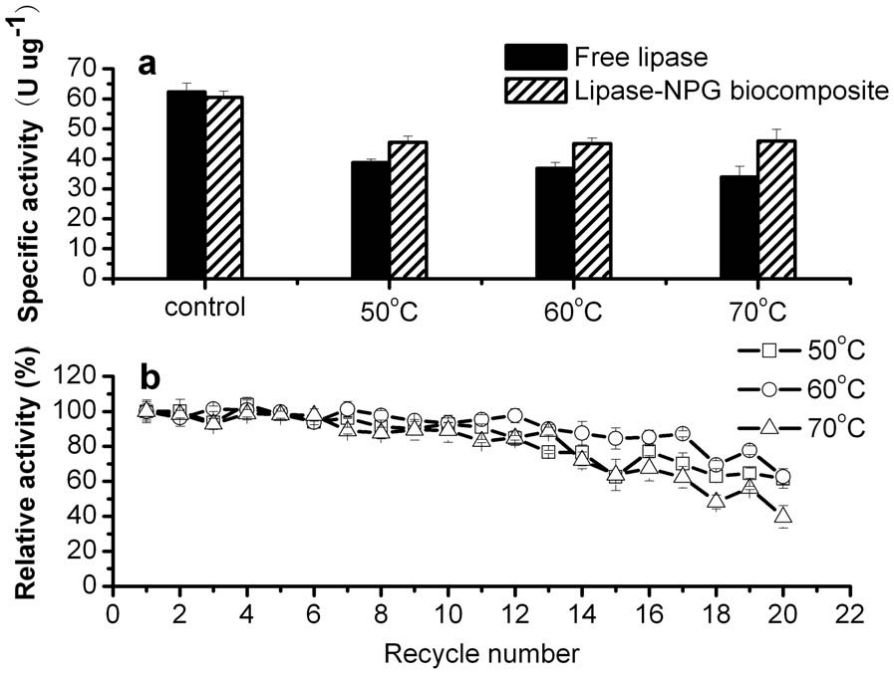
Tolerance stabilities of free lipase and the lipase-NPG biocomposite to heat (a), and reusability of the lipase-NPG biocomposite after treated by heat (b).

The risk of denaturation caused by heat could be effectively alleviated by using the lipase-NPG biocomposite as shown in Figure 5. After incubation in the temperature range from 50 to 70°C for 30 min, only 62, 59, and 54% of initial activity were observed for free lipase (Figure 5a). Compared with free lipase, the lipase-NPG biocomposite presented evidently higher stability and better reusability. After treated by heat, the lipase-NPG biocomposite exhibited much enhanced stability with 75, 74, and 76% of activity retention for 50, 60 and 70°C, respectively. In addition, almost no decrease in catalytic activity was observed after 10 cycles for the lipase-NPG biocomposite upon pre-treatments by heat. After 20 cycles, 61, 62, and 40% of activity remained for the lipase-NPG biocomposite incubated at 50, 60, and 70°C, respectively (Figure 5b).

**Figure 6:**
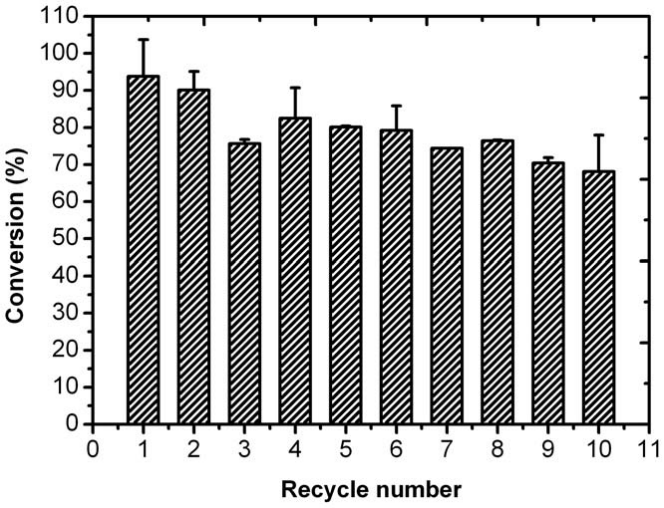
Catalytic conversion of soybean to biodiesel by the lipase-NPG biocomposite.

In order to further demonstrate the excellent performance of the lipase-NPG biocomposite, transesterification reaction for biodiesel production was investigated. The conversion of soybean oil to biodiesel by free lipase was 74% at 24 h, while the lipase-NPG biocomposite displayed even higher activity and superior stability in this reaction as shown in Figure 6. During the first two cycles, the conversion rate maintained an impressive high value of more than 90%. After 10 cycles, the conversion rate was still over 68%.

## Discussion

The aim of enzyme immobilization is to maintain the catalytic activity whilst improving its stability and ease of reuse. In this study, we constructed the enzyme-NPG biocomposites by assembling various enzymes onto the surface of NPG. It was clearly observed that the resulted enzyme-NPG biocomposites demonstrated not only remarkable catalytic performance but also excellent reuse stability compared with free enzymes. These results are superior to those reported in literatures [14], [25], [26].

Excellent catalytic performance alone is not enough to ensure a high stability under different conditions. The stability of enzyme plays a key role during the practical use of enzyme. From above results, the enzyme-NPG biocomposites have presented remarkable catalytic performance. Lipase is one of the most utilized classes of biocatalysts [27], [28]. It can be widely used in the enzymatic organic synthesis and clinical analysis [28]. Denaturation of lipase, which destroys its catalytic activity and stability, can be induced by heat or organic solvents. Thus, the lipase-NPG biocomposite was investigated under different experimental conditions. The results showed that the novel biocomposite possessed significantly enhanced reaction durability under various thermal and in organic solvent systems. In addition, a conservative estimation suggested that the lipase-NPG biocomposite could retain its high activity for at least 240 h in transesterification reaction system. These results were markedly better than previous reports where a significant decrease in activity was observed within 10 recycles in biodiesel production using immobilized lipase [14], [29]–[31]. The high conversion rate once again confirmed that NPG is an excellent support for enzyme immobilization.

**Figure 7:**
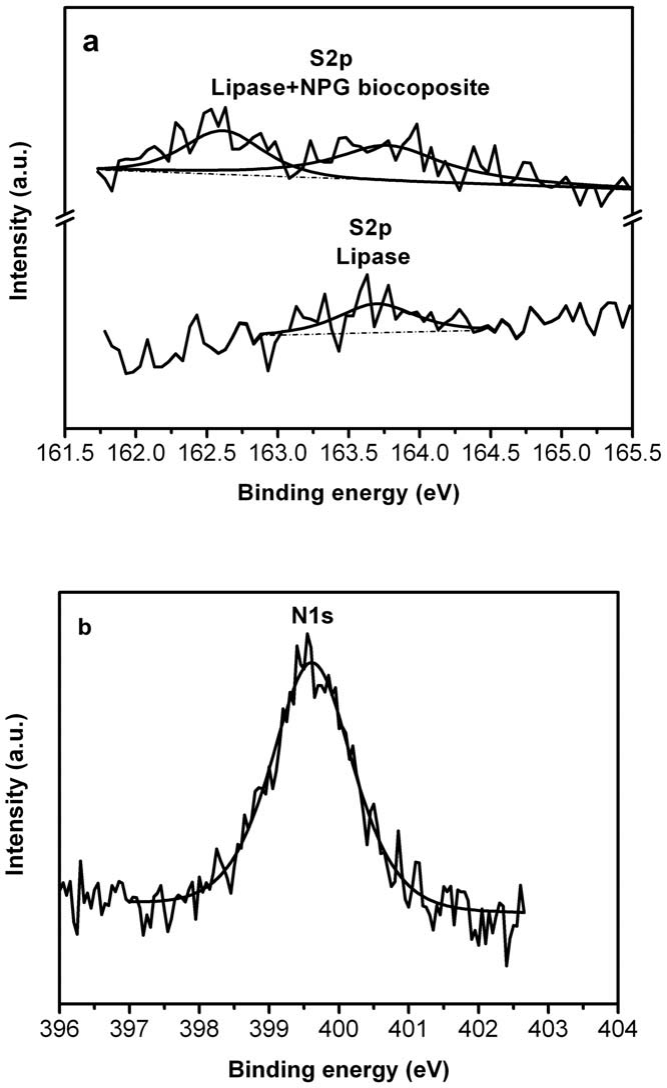
XPS spectra of S2p (a) for free lipase and the lipase-NPG biocomposite; XPS spectra of N1s (b) for the lipase-NPG biocomposite.

The above results clearly suggested that the immobilization using bio-compatible and highly conductive NPG could enhance the stability of enzymes. The excellent catalytic performance and stability of the enzyme-NPG biocomposites might be explained by their physical confinement inside the relatively small pores [1], [2], [32]. Especially, the size match between pore dimension and the molecular diameter of enzymes and the suitability of gold to function as an immobilization medium are of key importance in achieving high enzymatic stability [1], [5]. This involves the adsorption of the enzyme with its active site oriented away from the porous surface with little leaching yet sufficient mobility to retain catalytic activity [1], [5], [32], [33]. Moreover, it is well accepted that the interaction of nanoscale gold with NH_2_ was as strong as that with the commonly used SH [34], [35]. The lipase from *Pseudomonas cepacia* has fourteen lysine residues and six cysteine residues (data from http://web.expasy.org/protparam/). The covalent attachments of enzymes by the amino and mercapto groups onto the surface of NPG could prevent the enzyme leaching, and the curvatures of the porous surfaces could provide an ideal configuration for multipoint covalent attachments to global enzyme molecules, resulting in better operational stability [36]. To prove this, X-ray photoelectron spectroscopy was used to probe the chemical state of the surface for the lipase-NPG biocomposite. In free lipase, there is only a single broad peak at about 163.7 eV for S2p (Figure 7a). In contrast, after immobilization on NPG, it splits into two peaks with binding energy in the range from 162.0 to 165.0 eV corresponding to the chemical states of sulfur in Au-S and S-H, respectively [37]. Additionally, the N1s binding energy of nitrogen in protein at 399.6 eV was also observed in the lipase-NPG biocomposite as shown in Figure 7b, while no the signal of N1s was observed on the bare NPG. These results consist with other researchers' reports when lactate dehydrogenase encapsulated in poly nanofibers and fluorescein isothiocyanate-conjugated bovine serum albumin encapsulated in poly(ethylene glycol) and poly(*ε*-caprolactone) [38], [39]. These features make NPG an efficient support for chemical and biomolecular species.

In conclusion, a simple and efficient method for enzyme immobilization onto a nanoporous metal carrier was developed, and the resulted enzyme-NPG biocomposites showed extraordinary catalytic performance and stability. Compared with other existing supports for enzyme immobilization, the presented method has several advantages such as simple immobilization procedure, excellent catalytic activity, considerable enhanced operational stability, and ease of employment and recovery for reuse. These characteristics suggest that this approach provides a general strategy for the encapsulation of functional macromolecules into the pores of nanostructured materials and the resulted nano-biocomposite materials hold great potential in applications such as biosensing, molecular electronics, catalysis, and controlled delivery.
